# Psychosis in Neuro-Developmental Disorders: A Phenomenological Approach

**DOI:** 10.1192/bjo.2025.10334

**Published:** 2025-06-20

**Authors:** Ayomipo Amiola, Sreeja Sahadevan, Ignatius Gunaratna, Regi Alexander

**Affiliations:** 1 Hertfordshire Partnership University NHS Foundation Trust, Little Plumstead, United Kingdom; 2 Norfolk and Suffolk NHS Foundation Trust, Norwich, United Kingdom; 3 University of Hertfordshire, Hatfield, United Kingdom

## Abstract

**Aims:** Psychotic illnesses are more common in people with intellectual disabilities with rates as high as three times what is found in the general population. Making a diagnosis of psychosis in intellectual disability is complicated by various reasons such as communication difficulties, comorbidities, cultural differences, diagnostic overshadowing, and atypical presentation. The presence of comorbid Autism can further complicate the diagnostic process.

The clinical approach in diagnosing psychosis in people with intellectual disabilities must be based on a phenomenological assessment that aims to clarify in the patient, objective reality (that may include the “normal alternate” reality of neurodivergence) and the “loss of reality contact” observed in psychosis, from one another.

Our aim in this article is to illustrate phenomenologically the atypical nature of psychotic symptoms in people with neurodevelopmental disorders compared with the general population.

**Methods:** We analysed features of the mental state examinations of men admitted to the regional medium secure unit for men with neurodevelopmental disorders over the period of June 2021 and September 2024, identifying atypical descriptions of psychotic phenomena as indicated in the ICD–11 criteria.

**Results:** Over the study period, 16 men with psychosis and neurodevelopmental conditions were managed. The abnormal phenomena recorded included both classical descriptions similar to the general population and some atypical descriptions also.

In addition to bizarre, persecutory and grandiose ideas, other abnormal beliefs were reported as “bad thoughts”, and “paranoia”. Somatic, visual, and auditory hallucinations were also documented. Second- and third-person auditory hallucinations were experienced as external, located inside the head, or in some cases unclear. Abnormal thinking processes were described as “muddled thoughts” or “my head is screwed”. Clinicians also highlighted muddled conversation, disjointed thoughts, speaking in unfamiliar (non-existent) language, repetitive speech, minimal effective conversation, perplexity and loosening of associations. Negative symptoms were quite common including grossly disorganised behaviours such as agitation, combativeness, physical and sexual violence.

**Conclusion:** Core features of neurodevelopmental disorders may be misinterpreted as symptoms of psychosis and when psychotic phenomena are described atypically, clinicians may miss the diagnosis often with negative consequences for the patients.

